# Developing effective data visualization tools for nutrition: reflections on the design of a Nutrition Scorecard in Nigeria

**DOI:** 10.12688/gatesopenres.13319.1

**Published:** 2021-07-05

**Authors:** Yashodhara Rana, Gianni Dongo, Caroline Snead, Grace Agi, Oluwagbenga Sadik, Rebecca Heidkamp, Ahmad Abdulwahab

**Affiliations:** 1 Results for Development, 1111 19th Street NW, Suite 700, Washington, D.C., 20036, USA; 2 Nigeria Governors’ Forum, 51 Lake Chad Crescent, Maitama, Abuja, Nigeria; 3 Institute of International Programs, John Hopkins Bloomberg School of Public Health, 615 N. Wolfe Street, Baltimore, Maryland, 21205, USA

**Keywords:** Nutrition data visualization tool, data-driven nutrition accountability, data-driven nutrition advocacy

## Abstract

There has been a growing number of nutrition data visualization tools (DVTs) to monitor progress towards targets and encourage action. However, there are few documented examples of how to go about designing effective DVTs for nutrition-related audiences. In this Open Letter, we summarize reflections from collaborative efforts between the Nigeria Governors’ Forum (NGF) and the Data for Decisions to Expand Nutrition Transformation project (DataDENT) in 2019-2021 to design a sub-national nutrition scorecard that aims to hold Nigeria’s 36 Governors accountable to nutrition commitments. Our reflections add to an emerging body of work advocating for DVT design processes to develop a specific theory of change for how the DVT will influence target groups and achieve aims. Once the target audience is identified, it is important to create a strong engagement strategy to ensure that the DVT promotes constructive action. We also highlight the importance of identifying actionable indicators through participatory processes. We hope that these insights about collaborative DVT design can be applied by countries and institutions who want to develop similar tools to advance the nutrition agenda in their context.

## Introduction

**Figure 1.**
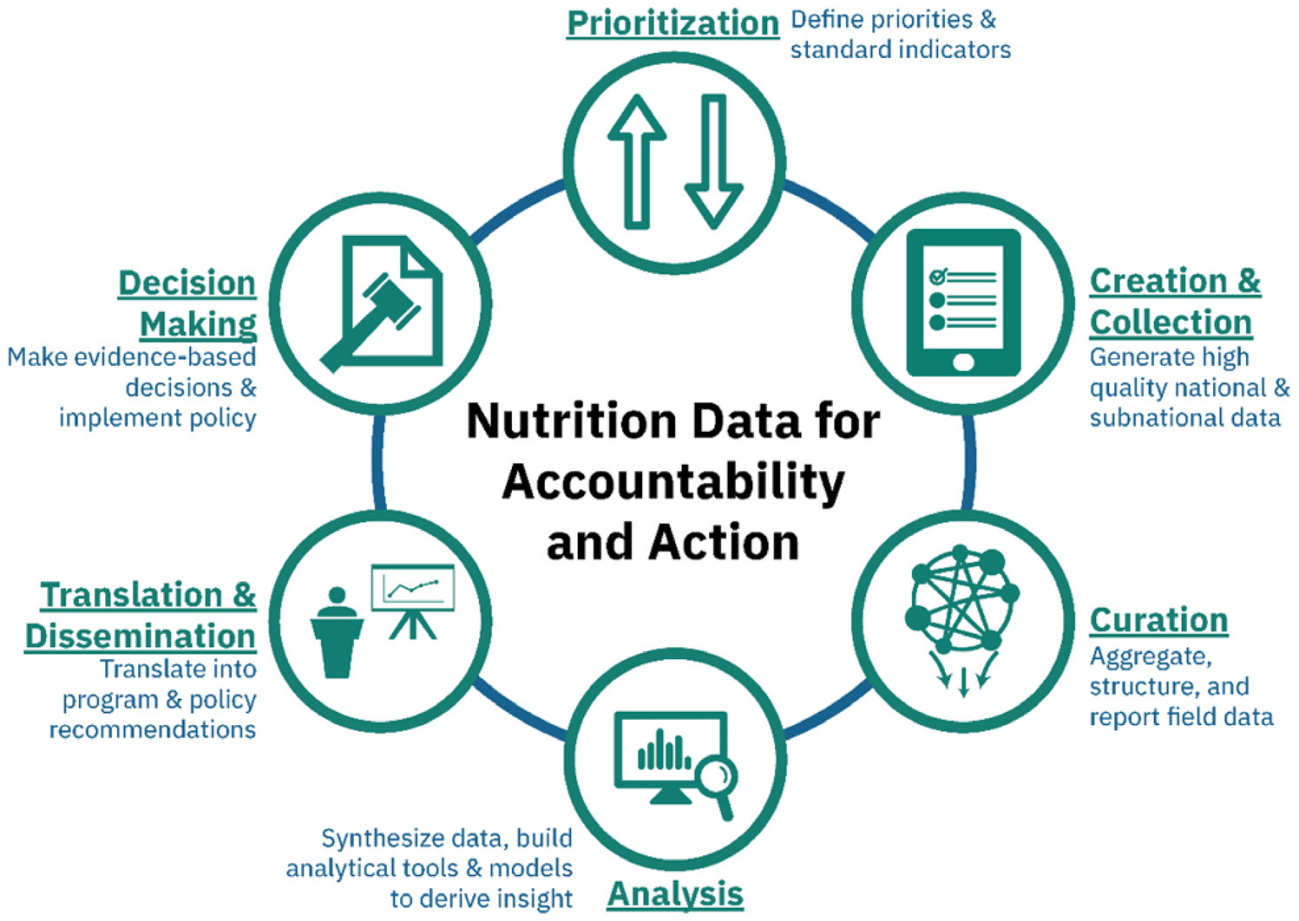
Nutrition data value chain. Adapted from Piwoz *et al.*, 2019 ^5^.

The 2014 Global Nutrition Report made a strong call for a nutrition data revolution so that governments can better utilize data to set their agenda, monitor progress, and ensure accountability for nutrition programs. This requires strengthening all links of the nutrition data value chain (DVC), including development of effective approaches for analysis and translation of key data for use by decision makers ( Figure 1) ^1^. In the last few years, the nutrition community has seen a proliferation of data visualization tools (DVTs) targeting audiences at global ^2^ and national levels ^3, 4^. However, many of these tools have not been designed with a clear user audience in mind nor an understanding of how the tool will be practically used as part of decision-making processes. There are few documented examples of how to go about designing effective DVTs for nutrition-related audiences.

In this article, we summarize reflections from collaborative efforts between the Nigeria Governors’ Forum (NGF) and the Data for Decisions to Expand Nutrition Transformation project (DataDENT) in 2019–2021 to design a sub-national nutrition scorecard that aims to hold Nigeria’s 36 Governors accountable to nutrition commitments. We first outline the purpose of the tool, then briefly describe the process we followed to develop it, and finally share reflections from the design process (summarized in Box 1). Our aim is to share insights about collaborative DVT design that can be applied by countries and institutions who want to develop similar tools to advance the nutrition agenda in their context.

**Box 1. Key reflections**

1. Start with a clear theory of change including an explicit understanding of who the target user group is and how the tool will influence their actions,
2. The purpose and audience dictate key features of the data visualization tool (DVT),
3. Directly engage target users in selecting or “co-creating” indicators as the process of indicator specification itself can influence policy design and implementation,
4. Select indicators that reflect progress in both the presence and quality dimensions of the commitments,
5. Consider both interim and aspirational indicators, when desired data are not available at design stage but feasible to collect over time,
6. Employ strategies to ensure data quality and build data ownership,
7. Ensure visualization features respond to the target audiences’ needs.
8. Create a strong engagement strategy with the target audience to ensure that the DVT promotes constructive action.

## Purpose of NGF’s Nutrition Scorecard

Improved nutrition is a key ingredient for the development of Nigeria’s rich human capital potential. Improvements in maternal and early childhood nutrition could yield $10 in economic returns for evey dollar invested through improved productivity and reduced healthcare costs over the lifespan ^6^. Nigeria is projected to have the world’s second largest working age population at the end of this century ^7^. However, at present, Nigeria has the second highest burden of stunted children in the world; nearly half of all deaths in Nigerian children under 5 years can be attributed to malnutrition ^8^. Nigeria’s 36 state Governors are well-positioned to advance the nutrition agenda by authorizing and supporting increased nutrition investment and providing oversight to nutrition activities. However, the Governors are faced with many competing priorities and demands for the available resources within their scope of influence. It is in this regard that the NGF Nutrition Scorecard was developed as an advocacy, accountability and monitoring tool for the nutrition commitments targeted towards achieving better nutrition outcomes. Launched in 2021, the scorecard is expected to be shared with the Governors at least semi-annually.

The Nigeria Governors’ Forum (NGF) is the non-partisan association of the 36 elected state Governors of Nigeria. Policy advisors who staff the NGF Secretariat provide administrative and technical support aligned with the forum’s vision to promote good governance and sustainable development. The NGF has a strong track record of using data to support decision-making and advocacy among Nigeria’s Governors. For instance, starting in 2009, the NGF designed and implemented state level scorecards to engage Governors around polio eradication efforts and to promote the establishment of state-supported health insurance schemes and release of basic health care provision funds ^9^. Given its successes deploying scorecards to promote Governors’ engagement on other issues, the NGF Secretariat decided to develop a similar tool for nutrition. The vision was for the scorecard to serve as an advocacy tool that will facilitate political commitment and investment in nutrition, promote Governor-level accountability and ultimately lead to improved nutrition outcomes across the states. 

DataDENT, an initiative funded by the Bill & Melinda Gates Foundation and focused on strengthening global and national DVC for nutrition, partnered with the NGF for the scorecard design process, building from experience landscaping users and producers of nutrition DVTs reaching global and Indian audiences, respectively ^2, 10^.

## Process for Nutrition Scorecard development

**Figure 2.**
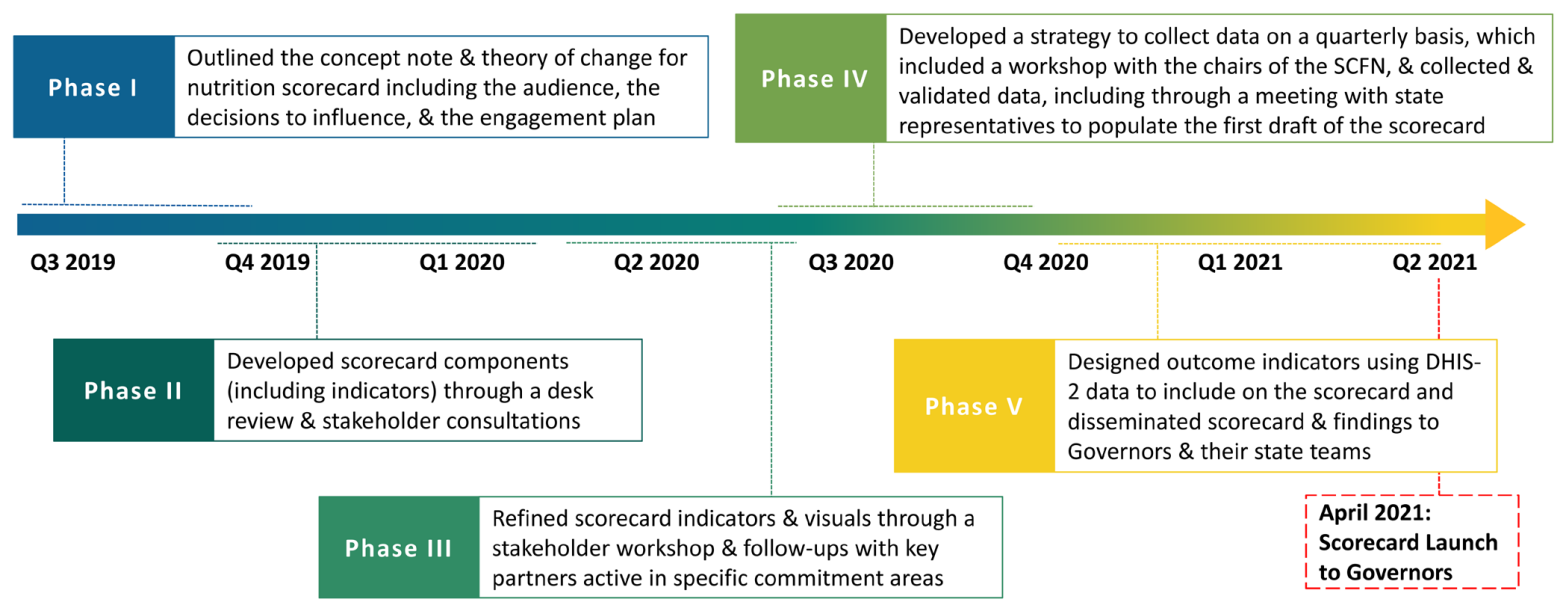
Nutrition Scorecard development process. SCFN=State Committees on Food and Nutrition; DHIS-2=District Health Information System 2.

In Figure 2, we briefly describe the five key phases of the scorecard development process.

The **first phase (Sep 2019 - Oct 2019) focused on conceptualization** where the design team ^a^, led by the health team of the NGF Secretariat, agreed on the overall theory of change for the nutrition scorecard. To support these efforts, the team systematically identified and actively engaged state and federal levels stakeholders who are key players positioned to drive state-level nutrition action. These included:

• **Governors and deputy Governors**: The design team engaged specific Governors who were known to champion nutrition and sought feedback from them about their experience with NGF scorecards on other thematic areas. Engaging early on with members of the primary user group helped give the project traction. 

• **Federal and state-level multi-sector nutrition coordination bodies**: In Nigeria, the National and State Committees on Food and Nutrition (SCFN) established by the Nigeria’s National Policy on Food and Nutrition, as well as the Federal Ministry of Finance, Budget, and National Planning are the primary stakeholders who coordinate cross-sector nutrition implementation in their jurisdictions.

• **Development partners**: Through the Nigeria Nutrition Partners Forum, the design team engaged leading development partners working in nutrition including UNICEF, Alive & Thrive, Save the Children, World Bank Accelerating Nutrition Results in Nigeria (ANRiN) project, and Civil Society – Scaling Up Nutrition in Nigeria (CS-SUNN). The early involvement of these influential stakeholders helped steady the project course and provided a broad range of technical resources available to the team during indicator development.

**Figure 3.**
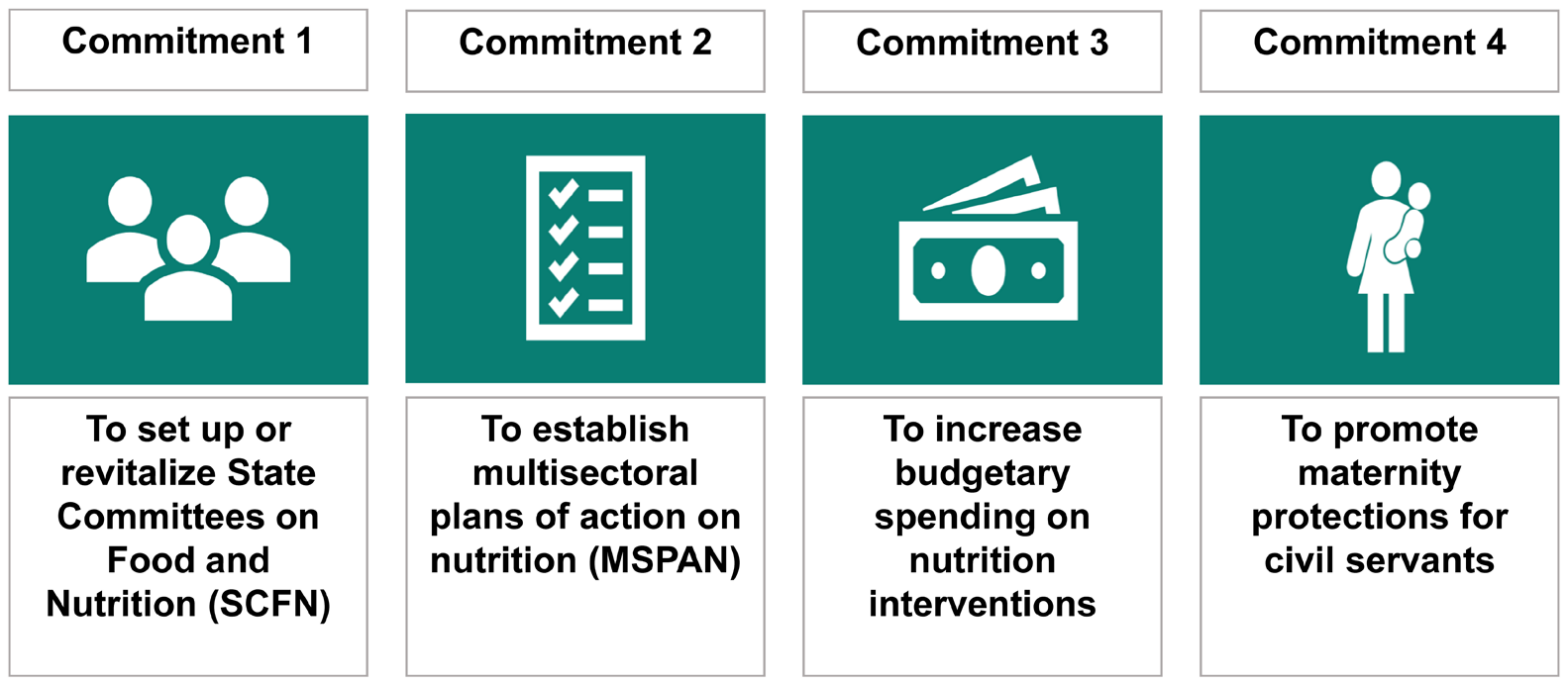
Nigeria Governors’ Forum commitments in nutrition.

The **second phase (Nov 2019 - Feb 2020) focused on development of key scorecard components including indicators** for the core nutrition commitments that the NGF had already identified as most directly shaping the enabling environment for nutrition in Nigeria ( Figure 3). The design team especially consulted Nigeria-based stakeholders (i.e., both state representatives and development partners) to unpack key challenges that states were facing in making progress on the four commitment areas that could be converted into indicators for inclusion in the scorecard. The team considered indicators that were meaningful, easily understood, time-bound, measurable, and regularly available.

The **third phase (end of Feb 2020 - Apr 2020) focused on refinement of the scorecard components**. A key activity was a participatory workshop with 30 federal and state-level government stakeholders and development partners. The workshop aims included 1) to present a revised draft of scorecard indicators based on earlier series of bilateral meetings with stakeholders, 2) to validate the draft indicators and arrive at a final set of indicators for the scorecard, 3) to familiarize a wider net of state actors with content of the scorecard for better ownership and seamless data sourcing/refreshing once the scorecard is launched, and 4) to identify additional uses for the scorecard. Post workshop, the design team revised indicators and followed up with select participants to ensure the revisions reflected the workshop inputs. Table 1 includes the final list of indicators included in scorecard’s first round of data collection.

**Table 1.** Nigeria Governors’ Forum nutrition indicators, scoring & validation methods, and inclusion in final scorecard for presentation to Governors. SCFN=State Committees on Food and Nutrition; MDA=Ministries, Departments, and Agencies; MSPAN=multisectoral strategic plan of action on nutrition.

Commitment	Indicator	Scoring Method	Validation Method	Included in Scorecard?
**To set up/revitalize SCFN**	SCFN held meeting in previous quarter (Jan - Mar 2020)	Yes – green No – red	SCFN last meeting minutes	**YES**
	Chairperson of SCFN chaired the previous quarter meeting	Yes – green No – red	SCFN last meeting minutes	**YES**
	Representatives from 5 nutrition relevant MDAs attended previous quarter meeting	Yes – green No – red (if any MDA is left out, the entire indicator is red)	SCFN last meeting minutes	**NO**
	SCFN has a costed workplan for the current year	Yes – green No – red	Copy of costed workplan	**NO**
	At least 75% necessary funding for workplan released quarterly	Yes – green No – red	Email verification from SCFN/state nutrition officer	**YES**
**To establish MSPANs**	MSPAN developed and costed; includes component activities from 2 nutrition-relevant MDAs	Yes – green (must be costed AND have activities from at least 2 MDAs) No – red	Copy of MSPAN	**YES**
	Approved MSPAN	Yes – green No – red	Copy of MSPAN	**YES**
	MSPAN progress reviewed annually	Yes – green No – red	Copy of meeting minutes from review meeting	**NO**
**To increase budgetary** ** spending on nutrition** **interventions**	State list of prioritized nutrition programs (from the MSPAN, if available) for budget tracking shared	Yes – green No – red	Copy of prioritized list	**NO**
**To promote maternity** **protections for civil** **servants**	6-month maternity leave exists	Yes – green No – red	Copy of circular	**YES**
	6-month maternity leave comes with full pay	Yes – green No – red	Copy of circular	**YES**
	Registered creche within the State Secretariat	Yes – green No – red	No validation currently required	**YES**
	State Secretariat creche is functional (ratio of 5 children to 1 nanny, handwashing station)	Yes – green (both the ratio of children to nannies and the handwashing station must be present) No – red	No validation currently required	**YES**
	State Secretariat creche has a breastfeeding/ complementary feeding corner	Yes – green No – red	No validation currently required	**NO**

The **fourth phase (May 2020 - October 2020) focused on collecting and validating state-level data to populate the scorecard.** The SCFN were identified as the most appropriate source for reporting on the scorecard indicators. The design team adapted to the coronavirus disease 2019 (COVID-19) pandemic lockdowns and conducted a virtual workshop with the chairpersons and secretaries of the SCFN. Aims included 1) understanding the challenges and opportunities that the SCFN Chairs foresee with establishing a functional SCFN in their respective states and solicit suggestions in response to these challenges; 2) creating a framework for how SCFN develop their annual workplans; and 3) familiarizing and generating buy-in for the NGF nutrition scorecard and to capture inputs on the scorecard data collection instrument. All 36 states submitted some data within five weeks of the workshop. The design team reviewed submissions and engaged each SCFN to validate results and follow-up on missing data. Prior to the presentation to the Governors, the design team also conducted a 1.5 hour virtual meeting with all state representatives and partners to 1) provide an overview of the findings and key takeaways from the first round of data population of the scorecard for feedback, and 2) explain how the NGF Secretariat intends to use the scorecard with the Governors and their teams.

**The fifth and final phase (end of October 2020 – April 2021) focused on designing outcome indicators and launching the scorecard.** The NGF sought to include a nutrition coverage or outcome indicator on the scorecard, in addition to the enabling environment indicators, to sensitize Governors to the nutrition situation in their respective states and motivate action. The Secretariat is rightly committed to using administrative data from the District Health Information System 2 (DHIS-2) to populate this indicator on the scorecard, given that it is updated more frequently than survey estimates and use of this data is necessary to prompt improvements in data quality. After a review of available indicators and data quality constraints, which included consultations with nutrition measurement experts and the DHIS-2 team in Nigeria, the design team selected severe acute malnutrition (SAM) treatment coverage as the nutrition coverage indicator to include on the scorecard ( Table 2). 

**Table 2.** Coverage Indicator Included on the Nigeria Governors’ Forum Nutrition Scorecard. SAM=severe acute malnutrition; U5=under 5 years.

Indicator	Definition	Source	Threshold
**U5 SAM Coverage**	% of children under five with SAM placed on treatment	District Health Information System 2 (Jan-Mar 2020)	75%

The scorecard was presented to the Governors at the meeting of the NGF in April 2021. The Governors acknowledged the importance of nutrition and the need to urgently address poor nutrition outcomes in the country. They agreed to follow up with their respective Commissioners of Health to galvanize appropriate actions to address identified gaps. Highlights of the scorecard were also shared with the Vice President of the Federal Republic of Nigeria, who serves as the chairman of the National Council on Nutrition. The NGF will continue to update the scorecard quarterly and present it to the Governors every six months.

## Key reflections

In the following section, we present key reflections based on the design process described above.

### Start with a clear theory of change including an explicit understanding of who the target user group is and how the tool will influence their actions

The “build-it-and-they-will-come” approach is insufficient. To ensure the use of data for decision-making, DVTs need an intentional theory of change about a) the target audience, b) clear decisions or behavior that the DVT is trying to influence, and c) a pathway of how the data/actions lead to change. Given this learning from the team’s landscape review of global nutrition DVTs ^2^, the design team had several rounds of discussions to specify the primary scorecard users: the Governors and their support staff at the onset of phase I. The team envisioned that the Governors would review the updated scorecard during a brief slot in the regular NGF quarterly meeting agenda. The guiding theory of change was that by comparing progress across states, the scorecard would motivate the Governors to maintain a good ranking by taking actions towards the commitments.

### The purpose and audience dictate key features of the DVT

For the NGF use case, the purpose and audience dictated two key choices: the type of DVT and the types of indicators. The design team chose a scorecard over other types of DVTs (e.g. dashboard; profile) because it was familiar to the Governors and would best facilitate cross-state comparisons of performance. The design team kept the scorecard focused on the four commitments that the Governors have the power to influence and that also have a conceptual linkage to improved nutrition outcomes. The commitments are focused on creating “enabling environments” for other stakeholders to implement effective nutrition programs. In turn, the indicators tracked under each commitment were selected to be “actionable” by the Governors. Rather than tracking quarterly changes in implementation inputs or coverage of specific nutrition interventions (which would be actionable indicators for other implementation-focused audiences), the indicators for the NGF audience are tied to policy, coordination, and budget.

### Directly engage target users in selecting or “co-creating” indicators as the process of indicator specification itself can influence policy design and implementation

The choice of indicators plays a powerful role in determining how people conceptualize a policy or program, what gets assessed, and consequently elevated for attention ^11^. While the commitment areas had been identified before DVT design phase I, the indicators to monitor those commitments had not. By engaging state-level representatives and development partners in indicator selection discussions, current barriers to progress on these commitments were identified. For example, discussion of the proposed indicator for “quarterly meetings of the SCFN with the Chairperson in attendance” revealed that these meetings were not happening and needed Governor attention. In addition, as indicators can influence user priorities, it was important to engage other coalitions involved with related issues to ensure the DVT indicators complement rather than compete with their ongoing efforts. Here, it is important to note that, in the case of the NGF Nutrition Scorecard, the design team developed unique fit-to-purpose indicator definitions, but it is preferable, whenever feasible, to align DVT indicators with established national and/or global standard definitions. 

### Select indicators that reflect progress in both the presence and quality dimensions of the commitments

Indicators can measure whether a critical component of the enabling environment exists, (e.g. whether a multi stakeholder platform exists; whether a legislation has been passed) without reflecting the quality or effectiveness of their implementation. Such indicators provide policymakers and advocates with limited information and, in some cases, a false notion that progress is being made ^12^. For this reason, the design team selected indicators that reflected progress in both the presence and quality dimensions of commitments. For example, states progress on the state level multisectoral nutrition plan commitment required that the plans were both multisectoral (i.e. included the participation of at least two nutrition related government ministries and agencies) and costed. An indicator under the SCFN commitment specified the quarterly release of at least 75% of necessary funding for the SCFN workplan because state-level stakeholders identified lack of funding as a key barrier to SCFN functioning. 

### Consider both interim and aspirational indicators, when desired data are not available at design stage but feasible to collect over time

There was no existing system to collect data for the proposed indicators for the four commitments, but it was feasible for the SCFN, with some initial support, to start to report on three of them. However, in the case of the nutrition spending commitment, most states did not have nutrition budget lines at the time of scorecard launch that could facilitate reporting. Many states did not even have costed multisectoral plans that identify which programs were relevant to track for nutrition spending. Therefore, for this commitment, the design team identified an interim indicator which assessed whether states had taken the first step of identifying which programs to track for nutrition spending. The design team then began engaging state actors and partners regarding how to develop the necessary state-level finance tracking systems to collect data on possible aspirational indicators (i.e. total nutrition spending per child under five) and drew attention to the need to collect this data. 

### Employ strategies to ensure data quality and build data ownership

Since the scorecard relied on self-reported data from the SCFN, the design team took several measures to ensure accurate reporting including hosting a virtual workshop with the chairpersons and secretaries of the SCFN to identify potential challenges with filling out the data collection questionnaire and ways to strengthen reporting capacity. After the workshop, the NGF team started a WhatsApp group with the SCFN representatives to facilitate reporting reminders, answer questions, and disseminate relevant information and events. For the first round, the NGF team was able to solicit responses from all 36 states within five weeks of the questionnaire being launched. The NGF team also asked for documentation to validate the SCFN self-report whenever feasible and appropriate ( Table 1). For example, those reporting that the SCFN met in the last quarter (Jan - March 2020) were also asked to share meeting minutes to confirm who specifically attended the meeting (scorecard indicator required that the chairperson of the SCFN attend the meeting). Such validation was essential to ensure accuracy and consistency of data across states. The SCFN were able to review their scorecard values before the presentation to Governors. 

### Visualization features should respond to the target audiences’ needs

**Figure 4.**
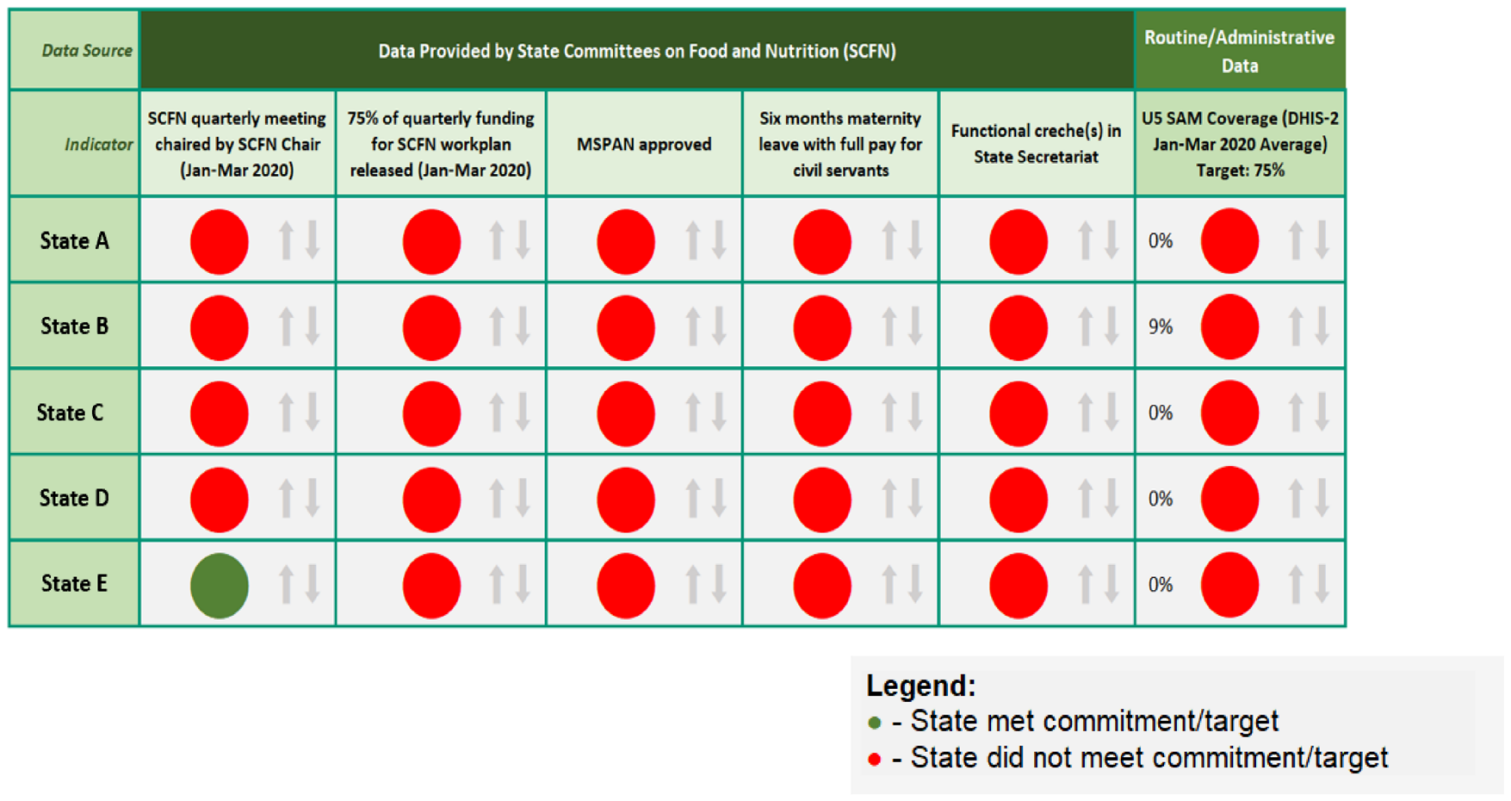
Final visualization format for the Nigeria Governors’ Forum nutrition scorecard. Green=State met commitment/target; Red=State did not meet commitment/target; MSPAN=multisectoral strategic plan of action on nutrition; DHIS-2=District Health Information System 2.

**Figure 5.**
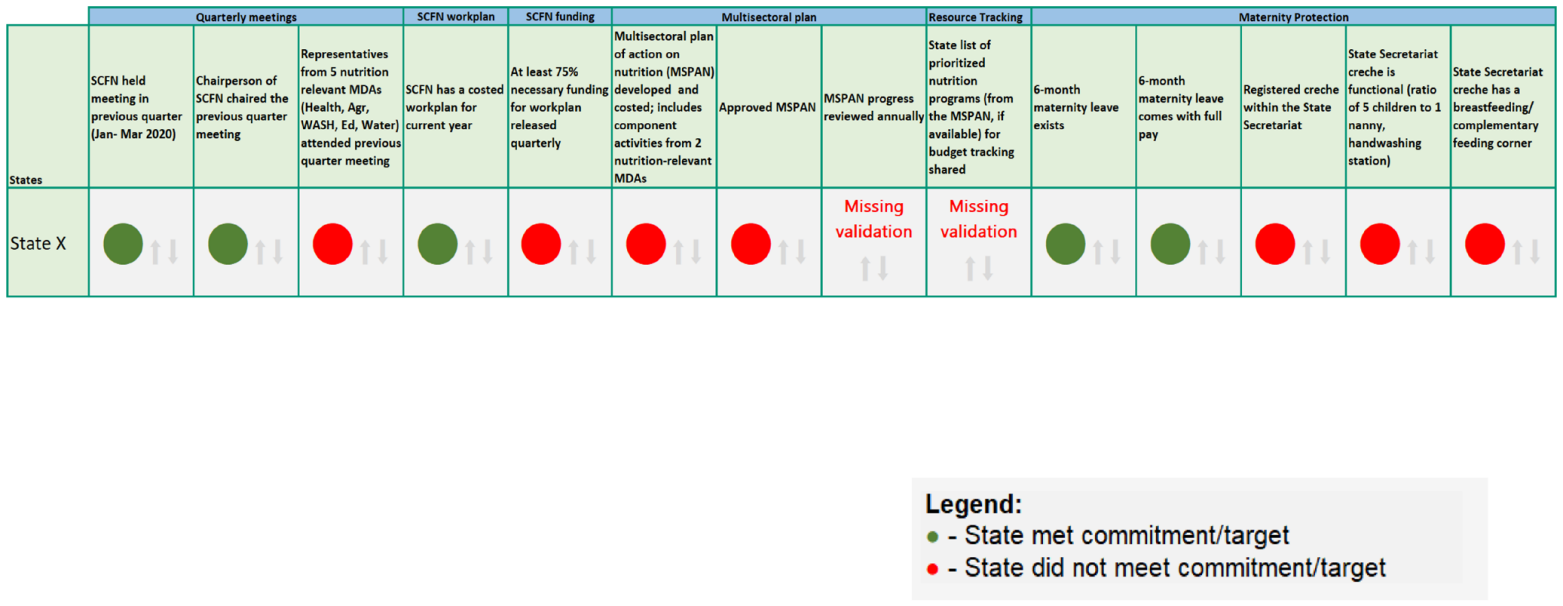
Full scorecard for State Committee on Food and Nutrition (SCFN)internal tracking purposes. Green=State met commitment/target; Red=State did not meet commitment/target.

It is important to tailor the visualization format to the needs of the intended audience. For the NGF Nutrition Scorecard, the design team gathered feedback on the Governors’ experience with other NGF scorecards. Feedback included that Governors do not have a lot of time to review the DVT in detail and prefer a simple visualization format that helps them assess their state’s progress over time compared to other states. The design team condensed the scorecard in a single page with fewer key indicators and, similar to earlier scorecards, used basic red vs. green color coding to show whether the indicator had been achieved. In addition, the design team also included a coverage indicator – SAM treatment coverage - in the scorecard and presented survey data on stunting and exclusive breastfeeding separately so that Governors had an eye on the overall state of nutrition in their respective states while assessing their progress on the enabling environment commitments. In future quarterly updates, cells will also include arrows to indicate direction of progress since the last presentation ( Figure 4). A second expanded version of the scorecard with the full set of indicators was developed for the SCFN to meet their internal tracking needs ( Figure 5).

### Create a strong engagement strategy with the target audience to ensure that the DVT promotes constructive action

In addition to the quarterly NGF meetings, the design team worked closely with nutrition stakeholders to develop guidelines that clearly lay out the actions required at state level to achieve each commitment and a roster of federal and state-level partners active in each commitment area who can provide Governors and the SCFN with technical assistance. The NGF Secretariat can use these resources to productively engage with Governors’ offices and SCFN in follow up to quarterly scorecard presentations. 

## Discussion

To our knowledge, this is one of the first studies to share reflections stemming from the design phase of a DVT that aims to influence nutrition-related decision making in low- or middle-income countries. Our reflections add to an emerging body of work advocating for DVT design processes to develop a specific theory of change for how the DVT will influence target groups and achieve aims ^2, 3, 13^. This is in contrast to the “if we build it, they will come” approach that is often used to justify DVT development. We hope it also provides practical insights to DVT producers about the importance of identifying actionable indicators through participatory processes.

The influence of the NGF nutrition scorecard is yet to be seen. In the next phase of work, the NGF and DataDENT design team will engage in rapid learning cycles to get Governor feedback and further adapt the scorecard. We plan to evaluate the impact of the scorecard and share findings with the global community.

## Data availability

No data are associated with this article.

## Footnotes

^a^The scorecard design team was led by the health team at the NGF Secretariat and supported by the DataDENT project
