# Supplementary material for: Developing effective data visualization tools for nutrition: reflections on the design of a Nutrition Scorecard in Nigeria
Source: Gates Open Res. 2021 Jul 5;5:98. [Version 1] doi: 10.12688/gatesopenres.13319.1 (PMC8609191; doi:10.12688/gatesopenres.13319.1)
Supplement: Peer review history

# Peer review: Reviewer response for version 1

**DOI:** 10.21956/gatesopenres.14562.r31300

**Published:** 2021-11-22

**Authors:** Kendra Siekmans

**Affiliations:** 1 Independent Consultant, Public Health Nutrition, Guelph, ON, Canada

Yashodhara Rana and colleagues provide a useful overview of their experience in designing a scorecard targeted to Nigeria's State Governors and State Committees on Food and Nutrition. They describe the purpose of the scorecard, the underlying theory of change informing its design, and the steps taken in the design process. The article ends with a list of reflections on their learning through the process, providing helpful guidance to those who may want to replicate such a process. While no information is provided about follow-up actions taken by the scorecard's target audience since the launch, the authors note that there are plans in place for learning processes and an evaluation of the scorecard's impact in the future. Thus, although the article is useful as an example of what a user-designed process looks like, there is no evidence yet of whether the theory of change will hold true and if/how the scorecard will contribute to increased investment and action for nutrition.

 Based on recent reviews of country experiences with nutrition technical assistance (see references 1 to 5), there is a need for context-specific tools to assist national and sub-national government stakeholders in monitoring progress for nutrition. It is commendable that the process described here was led by a national design team that continues to be involved and has a defined role in preparing and disseminating the scorecard to the Governors on a quarterly basis. The ongoing engagement with State Committees on Food and Nutrition and plans for providing technical assistance are expected to be important for building state-level capacity and supporting the system changes needed.

Does the article adequately reference differing views and opinions?

Yes

Are all factual statements correct, and are statements and arguments made adequately supported by citations?

Yes

Is the Open Letter written in accessible language?

Yes

Where applicable, are recommendations and next steps explained clearly for others to follow?

Yes

Is the rationale for the Open Letter provided in sufficient detail?

Yes

Reviewer Expertise:

nutrition programme monitoring and evaluation, information systems for nutrition, enabling environment for nutrition, nutrition technical assistance

I confirm that I have read this submission and believe that I have an appropriate level of expertise to confirm that it is of an acceptable scientific standard.

## References

[1] Measuring Progress: Summary of Progress Assessments of Eight Technical Assistance Assignments from 2017-2019 by NTEAM’s TAN Project. *Nutrition International* .2020; Reference source

[2] : External Progress Assessment of Technical Assistance Delivered under Nutrition International’s TAN Project. *Nutrition International* .2021; Reference source

[3] Providing technical assistance for SUN countries: Documenting the added value of Nutrition International and MQSUN technical assistance for Nutrition in eight SUN countries. *ENN* .2020; Reference source

[4] : Designing Effective Multisectoral Nutrition Collaboration: Insights and Lessons Learned from the National Information Platforms for Nutrition International Partnerships. *National Information Platforms for Nutrition* . Reference source

[5] Effectiveness of Technical Assistance: Synthesis Report on Key Findings from Six Case Studies on the Effectiveness of Technical Assistance Provided by the TAN MQSUN Facility 2017-2020. *TASC* .2021;

# Peer review: Reviewer response for version 1

**DOI:** 10.21956/gatesopenres.14562.r30870

**Published:** 2021-07-19

**Authors:** Melanie Renshaw

**Affiliations:** 1 African Leaders Malaria Alliance (ALMA), Nairobi, Kenya

The report is clear and well written. I agree with the need for simplicity but would include a 'yellow' for some progress to encourage the decision makers that there has been progress. It is not clear how actions will be captured and progress against the actions tracked, so perhaps more details on this. ALMA has created a scorecard hub which documents scorecard best practices and includes 'how to' guidelines,as well as including country uploaded scorecards - this could perhaps be considered as a reference? ( https://scorecardhub.org/). Finally, it would be good to understand the multisectoral dimension including how data across sectors will be systematically collected to allow automatic data population over time.

Does the article adequately reference differing views and opinions?

Yes

Are all factual statements correct, and are statements and arguments made adequately supported by citations?

Yes

Is the Open Letter written in accessible language?

Yes

Where applicable, are recommendations and next steps explained clearly for others to follow?

Yes

Is the rationale for the Open Letter provided in sufficient detail?

Yes

Reviewer Expertise:

ALMA has supported over 40 countries in the roll out of country scorecards and accountability tools including for malaria, RMNCAH, nutrition, NTDs and community scorecards

I confirm that I have read this submission and believe that I have an appropriate level of expertise to confirm that it is of an acceptable scientific standard.
